# Virulence factors and antimicrobial resistance of uropathogenic *Escherichia coli* (UPEC) isolated from urinary tract infections: a systematic review and meta-analysis

**DOI:** 10.1186/s12879-021-06435-7

**Published:** 2021-08-04

**Authors:** Gabriel Kambale Bunduki, Eva Heinz, Vincent Samuel Phiri, Patrick Noah, Nicholas Feasey, Janelisa Musaya

**Affiliations:** 1 Department of Pathology, Kamuzu University of Health Sciences (former College of Medicine/University of Malawi), Blantyre, Malawi; 2 Africa Centre of Excellence in Public Health and Herbal Medicine (ACEPHEM), Blantyre, Malawi; 3 Malawi-Liverpool-Wellcome Trust Clinical Research Programme, Blantyre, Malawi; 4 Department of Infectious Diseases, Faculty of Medicine, Université Catholique du Graben, Butembo, Democratic Republic of the Congo; 5 Liverpool School of Tropical Medicine, Pembroke Place, Liverpool, L3 5QA UK; 6 Department of Public Health, Kamuzu University of Health Sciences (former College of Medicine/University of Malawi), Blantyre, Malawi; 7 Department of Surgery, Queen Elizabeth Central Hospital, Blantyre, Malawi

**Keywords:** Urinary tract infection, *Escherichia coli*, Virulence factors, Antimicrobial resistance, Systematic review, Meta-analysis

## Abstract

**Background:**

Uropathogenic *Escherichia coli* (UPEC) are amongst the most frequent causes of urinary tract infections. We report a systematic review and meta-analysis of virulence factors and antimicrobial resistance of UPEC isolated from urinary tract infections.

**Methods:**

A systematic review and meta-analysis were performed using PRISMA guidelines (Research Registry ref. 5874). Data were extracted from PubMed/MEDLINE and ScienceDirect databases for studies published from January 1, 2000 to December 31, 2019. Studies reporting antimicrobial resistance and virulence factors of UPEC isolated in confirmed urinary tract infections (≥10^5^CFU/ml) were eligible. Prevalence of antimicrobial resistance and virulence factors of UPEC were estimated using random-effects meta-analysis model. Estimates with 95% confidence intervals, I-square (*I*^2^) statistic, and Cochran’s Q test were computed using the score statistic and the exact binomial method by incorporating the Freeman-Tukey double arcsine transformation of proportions.

**Results:**

Our search returned 2504 hits, of which 13 studies were included in the meta-analysis, totalling 1888 UPEC isolates. Highest antimicrobial resistance rates were observed among the antibiotic class of tetracycline in 69.1% (498/721), followed by sulphonamides in 59.3% (1119/1888), quinolones in 49.4% (1956/3956), and beta-lactams in 36.9% (4410/11964). Among beta-lactams, high resistance was observed in aminopenicillins in 74.3% (1157/1557) and first generation cephalosporins in 38.8% (370/953). Meanwhile, virulence factors with highest prevalence were immune suppressors (54.1%) followed by adhesins (45.9%). Taken individually, the most observed virulence genes were *shiA* (92.1%), *CSH* (80.0%), *fimH/MSHA* (75.3%), *traT* (75.1%), *sisA* (72.2%), *iucD* (65.7%), *iutA* (61.8%), *kpsMTII* (60.6%), and *PAI* (55.2%).

**Conclusions:**

The increased antibiotic resistance of UPEC isolates was demonstrated and suggested a need for reassessment of empirical therapies in urinary tract infections treatment caused by this pathogen. In addition, this pathotype exhibited diverse surface and secreted virulence factors.

**Supplementary Information:**

The online version contains supplementary material available at 10.1186/s12879-021-06435-7.

## Background

Antimicrobial resistance (AMR) has increasingly been reported in bacteria causing urinary tract infections (UTI) during the last few decades and has become a major public health concern [1]. Globally the most common cause of UTI is *Escherichia coli* [2], a ubiquitous gram negative pathogen and member of the family *Enterobacteriaceae*. Uropathogenic *E. coli* (UPEC) are among the most common extra-intestinal pathogenic *E. coli* (ExPEC) encountered [3]. *E. coli* typically acquires AMR genes through mobile genetic elements (MGE), such as plasmids, insertion sequences, transposons, and gene cassettes/integrons [4]. A large number of resistance-encoding mobile elements, in particular plasmids, are shared between different members of the *Enterobacteriaceae* and thus further promote the spread of resistance genes [5]. MGE can also encode for virulence factors, and there may be interplay between virulence and antimicrobial resistance [4].

*E. coli* is a commensal inhabitant of human and animal gastrointestinal tract and maintains the stability and homeostasis of luminal microbial flora by the symbiotic interplay with its hosts [6]. While confined in the intestinal lumen, this bacterium remains harmless in healthy individuals but some strains may cause diarrhoea in some circumstances. Meanwhile, several *E. coli* lineages have acquired specific virulence characteristics, giving them the capacity to thrive in specific niches and cause disease generally grouped in three clinical syndromes: enteric/diarrhoeal disease, urinary tract infections (UTIs) and sepsis/meningitis [7]. These virulence characteristics are often encoded on genetic elements that can be mobilized to establish new combinations of virulence factors in different strains, or on genetic elements that have once been mobile but now become fixed in the chromosome [7]. UPEC has large and small pathogenicity islands (PAIs), which are integrated mobile elements that encode for the key virulence factors. These allow UPEC to infect an immunocompetent host, as they encode for factors enabling it to colonize the periurethral area and ascend the urethra to the bladder [7].

Key virulence factors involved in the pathophysiology of UTIs function in invasion, colonization and mediation of host defences subversion [8]. PAIs furthermore often carry cryptic or functional genes that encode mobility factors, such as integrases, transposases and insertion sequence elements [7], which are traces from their mobile history and may promote and contribute to the spread and emergence of antimicrobial resistance [9–12].

Community and hospital acquired UTIs significantly affect the life quality of infected patients [13]. It has been reported that *E. coli* is expected to cause loss of lives of more than 3 million people each year by 2050 following the increase in multi-drug resistance. A particular focus is placed to track carbapenem-resistant strains which are spreading world-wide and only leave few last-line treatment options like colistin or tigecycline, which are known for severe side-effects and not applicable for all types of bacterial infections due to reduced tissue permeation, respectively; and resistance mechanisms against both of these are increasingly observed [14].

Here, we report a systematic review and meta-analysis of virulence factors and antimicrobial resistance of UPEC. We also briefly review the relationship between virulence factors and antimicrobial resistance.

## Methods

The preferred reporting items for systematic reviews and meta-analyses (PRISMA) guidelines [15] were used in conducting this systematic review. The protocol of this review was registered in the Research Registry (ref 5874) (https://www.researchregistry.com/browse-the-registry#home/registrationdetails/5f2bbf5e83bd1d0017e9ec9e/).

### Search strategy

The electronic bibliographic databases PubMed/MEDLINE and ScienceDirect were searched in all fields with the search terms combined as follow: Virulence factors OR virulence AND factors OR virulence factors AND associated AND anti-infective agents OR anti-infective agents OR anti-infective AND agents OR anti-infective agents OR antimicrobial AND resistance AND uropathogenic *Escherichia coli* OR uropathogenic AND *Escherichia* AND *coli* OR uropathogenic *Escherichia coli* AND UPEC.

A 20 year time period, between 2000 and 2019, was considered for the search. This time limit was based on possible changes in the virulence, microbiology, epidemiology and antimicrobial susceptibility patterns of uropathogenic *E. coli* [16]. The number of records retrieved for each database searched was recorded. Reference lists of identified studies were checked manually to supplement the electronic search. Retrieved studies were exported into Mendeley Desktop version 1.19.4 and screened against inclusion and exclusion criteria.

### Inclusion and exclusion criteria

Observational (cross sectional, prospective and retrospective cohort, and case-control) studies reporting the virulence and antimicrobial susceptibility patterns of uropathogenic *E. coli* isolated from human samples from patients of any age and region were included in this review. Studies published before 2000 and after 2019, and those reporting results from animal samples were excluded. Grey literature was not considered. Studies published in any other language than English and those with non-accessibility to full-texts were excluded. Only studies reporting their microbiologically confirmed UTI (≥10^5^CFU/ml) using the Centre of Disease Control and Prevention’s definition were included in this review [17]. This review included both inpatients and outpatients with UTIs. Hence, data from a study which used both settings were considered as two separate studies and each was counted as a single study.

### Study selection

The identified titles and abstracts of all the studies retrieved in the electronic databases and searched manually were screened for their appropriateness and relevance to the main aim of the systematic review. Studies that were irrelevant were excluded at this stage. Full texts of potentially relevant studies were downloaded and added to a created Mendeley library and were assessed for inclusion and exclusion criteria of this systematic review. Quality and risk bias assessment was done for included studies containing relevant data for the systematic review and meta-analysis.

The author GKB performed the selection process and other stages of this review. Ten percent of identified studies were screened independently for inclusion and exclusion criteria by JM at each stage of the review. The discrepancies in either the decision on inclusion or exclusion of studies, quality assessment or on data extraction were discussed between GKB and JM to make the consensus for the final decision.

### Data extraction

Data extraction was independently done by GKB and JM and was compared for matching. For variables with missing information or with disagreement between the two authors, a consensus between the authors was made for the final decision.

An Excel 2010 spreadsheet was used for data extraction and contained the following data for studies that met inclusion criteria: first author, year of publication, country/place of study, study population/sample size, patient types (inpatients or outpatients), prevalence of antimicrobial resistance of different antibiotics tested, method used for detecting virulence factors, and prevalence of virulence factors.

### Quality assessment and risk of bias in individual studies

The Newcastle-Ottawa Scale (NOS) adapted for cross-sectional studies was used for assessing the risk of bias of included studies (Supplemental file 1). This scale was adapted from the NOS quality assessment scale for cohort studies. The assessment was in the area of selection (maximum of 3 points), comparability (maximum of 2 points) and outcome (maximum of 3 points). This was done by GKB and JM. Studies were classified into 4 categories: very good (9–10 points), good (7–8 points), satisfactory (5–6 points) and unsatisfactory (0–4 points). The complete assessment of studies is found in the supplemental file 2.

### Statistical analysis

We used *metaprop* and *metaprop_one* commands in Stata 16 for Windows to conduct the meta-analysis. Prevalence of antimicrobial resistance and virulence factors of UPEC were estimated using random-effects meta-analysis model. The 95% Wald confidence intervals were computed using the score statistic and the exact binomial method by incorporating the Freeman-Tukey double arcsine transformation of proportions for avoiding exclusion of studies with proportion equal to 0 or 1 from the calculation of the estimate [18]. The effect size of the prevalence was considered statistically significant when *p*-value was < 0.05. The proportions with 95% Wald confidence intervals were generated. I-square (*I*^2^) statistic test was used to evaluate the proportion of statistical heterogeneity and the Cochran’s Q test was used to explain the degree of heterogeneity. The funnel plot publication bias was not assessed as it is not relevant for the prevalence studies [19], however, the Egger’s linear regression test was used.

## Results

### Study selection

**Fig. 1:**
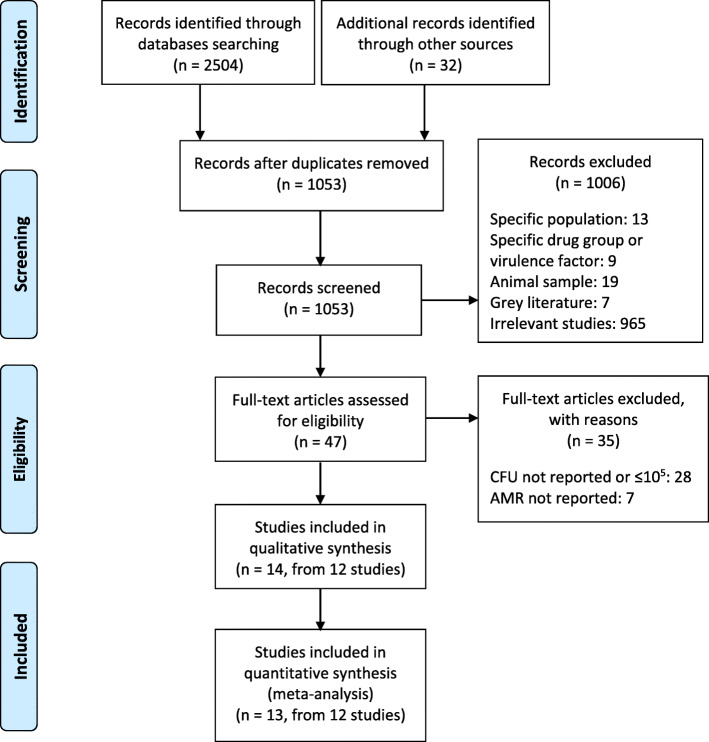
The PRISMA flowchart for literature search and study selection

The literature search using PRISMA identified a total of 2536 studies (2504 studies through databases searching and 32 from other sources). After removing duplicates, 1053 were screened for eligibility. After the screening of titles and abstracts, 1006 studies were excluded. Full-texts of the remaining 47 studies were read and 35 more studies were excluded. At the end, 12 papers were included; from which 14 studies were included in the qualitative analysis and 13 in the meta-analysis as explained below (Fig. 1).

### Study characteristics

Study characteristics of included studies are presented in Table 1. The 14 studies reported in this systematic review represent 8 countries, namely Iran (6 studies), China (2), India (1), Poland (1), Jordan (1), Mexico (1), Brazil (1) and Nigeria (1). The total sample size of UPEC isolates from the 14 studies is 1888 (range 32–227). Nine of the 14 studies report UPEC from inpatients [2, 20–23, 25, 27, 29, 30] while 5 are from outpatients [20, 24, 26, 28, 30]. Among the 14 studies, 2 studies reported UPEC from in- and out-patients [20, 30] and were therefore considered each as single study for each category of patients. Meanwhile, 2 other studies [26, 27] reported UPEC in in- and out-patients but did not specify sample size in each category of patients. After consensus of authors, one was considered as reporting in-patients [27] and another one out-patients [26]. Among the 13 studies included in the meta-analysis, one reported in- and out-patient UPEC but did not distinguish the two categories while reporting the antimicrobial resistance rate [20], and was hence considered as a single study in the meta-analysis.

**Table 1:** Characteristics of included studies after full assessment

Authors	Publication year	Country	Sample size	Type of patients	Method for VFs detection	NOS points
Ghazvini et al. (1) [20]	2019	Iran	168	Outpatients	PCR	8
Ghazvini et al. (2) [20]	2019	Iran	32	Inpatients	PCR	6
Jadhav et al. [21]	2011	India	150	Inpatients	Phenotypical	6
Kot et al. [22]	2016	Poland	173	Inpatients	Phenotypical, PCR	6
Malekzadegan et al. [23]	2018	Iran	126	Inpatients	PCR	8
Miranda-Estrada et al. [24]	2017	Mexico	107	Outpatients	Phenotypical, PCR	8
Neamati et al. [25]	2015	Iran	150	Inpatients	PCR	5
Oliveira et al. [26]	2011	Brazil	204	Outpatients	PCR	8
Olorunmola et al. [27]	2013	Nigeria	137	Inpatients	Phenotypical	5
Raeispour et al. [2]	2018	Iran	60	Inpatients	PCR	5
Shakhatreh et al. [28]	2019	Jordan	227	Outpatients	PCR	5
Tabasi et al. [29]	2015	Iran	156	Inpatients	Phenotypical	8
Wang et al. (1) [30]	2014	China	69	Inpatients	PCR	8
Wang et al. (2) [30]	2014	China	129	Outpatients	PCR	6

Of the 14 included studies, 9 studies used the polymerization chain reaction (PCR) as method for detecting virulence factors of UPEC [2, 20, 22–26, 28, 30], 3 studies used phenotypical methods [21, 27, 29], while 2 studies used both methods [22, 24].

### Quality assessment and bias assessment

Based on the quality assessment of studies using the NOS assessment, six studies scored 8 points [20, 23, 24, 26, 29, 30], which could be regarded as good studies. While eight studies scored 5–6 points [2, 20–22, 25, 27, 28, 30], and could be regarded as satisfactory studies. The detailed NOS assessment is found in the supplemental file 2. A bias assessment was done on the countries of origin of the included studies. The Egger’s regression intercept was of − 7.71, with a standard error of 2.23, 95% CI: − 2.26 – 3.46, t-value of 6.0 and *p* = 0.013. The fact that almost 50% of included studies in this meta-analysis came from a single country could have introduced a bias in the analysis.

### Antimicrobial resistance and virulence factors of UPEC

**Fig. 2:**
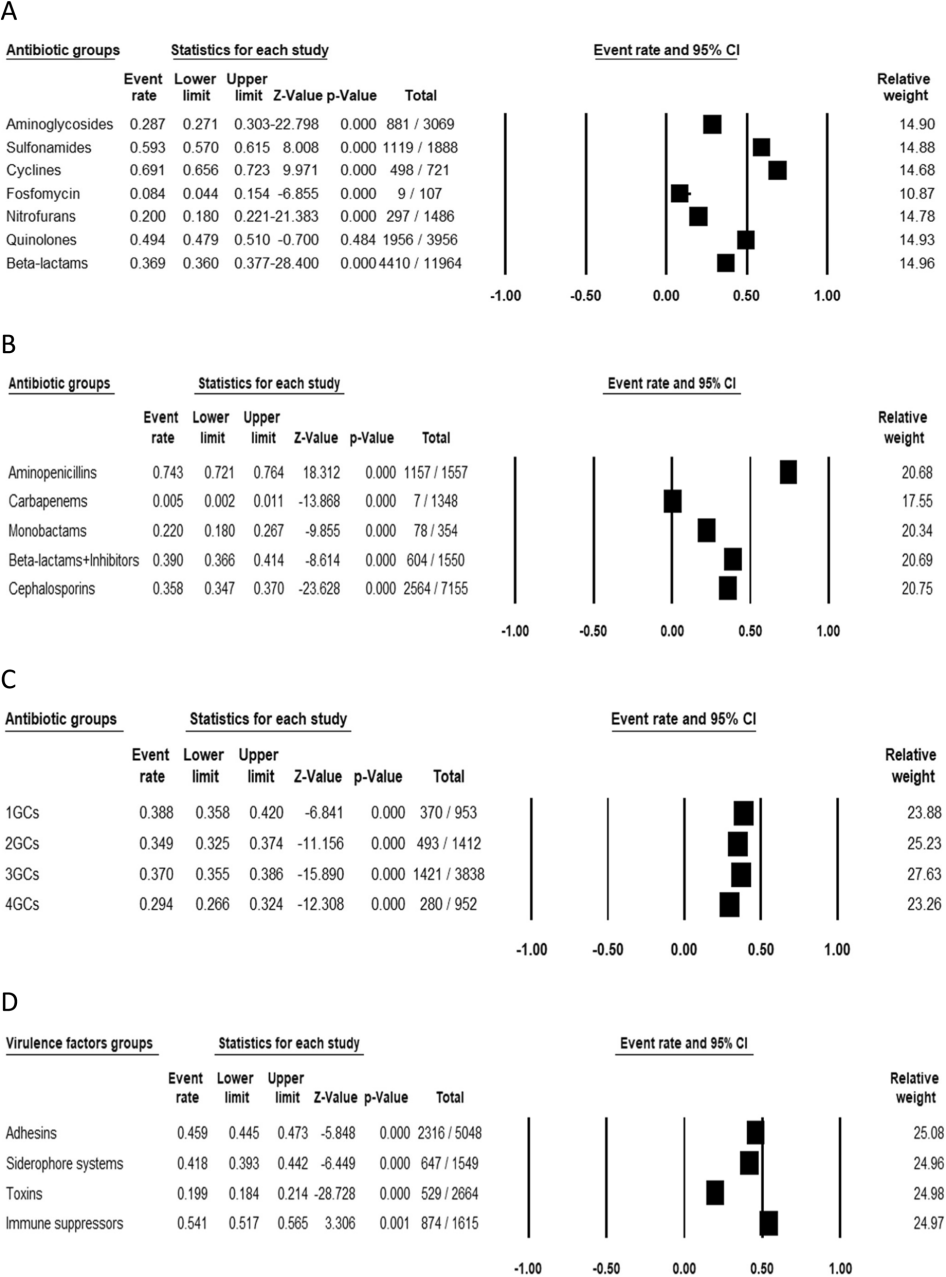
Forest plot of UPEC resistance to different antibiotic subgroups (**A** main antibiotic groups, **B** Beta-lactams classes, **C** Cephalosporins classes) and virulence factors groups (**D**)

Of the 13 studies included in the meta-analysis, the pooled number of *E. coli* isolates was 1888. Tables 2 and 3 both present the specific proportions of antimicrobial resistance and virulence factors with 95% exact confidence intervals for each antibiotic and virulence factor, and the *I*^*2*^ and Q statistics which describe proportions of total variations due to inter-antibiotics/virulence factors heterogeneities. The heterogeneity tests for both antimicrobial resistance and virulence factors were significant (*I*^*2*^ > 75%). Highest antimicrobial resistance rates were observed among the antibiotic class of tetracyclines in 69.1% (498/721) followed by sulphonamides in 59.3% (1119/1888), quinolones in 49.4% (1956/3956), beta-lactams in 36.9% (4410/11964), aminoglycosides in 28.7% (881/3069), nitrofurans in 20.0% (297/1486) and fosfomycin in 8.4% (9/107) (Fig. 2a). Among beta-lactams, high resistance was observed in aminopenicillins in 74.3% (1157/1557), beta-lactam associated with inhibitors in 39.0% (604/1550), cephalosporins in 35.8% (2564/7155) and monobactam in 22.0% (78/354). However, carbapenems had the least rate of resistance, 0.5% (7/1348) (Fig. 2b). Among the cephalosporins, high rates of resistance were observed in the first generation cephalosporins in 38.8% (370/953) and third generation cephalosporins in 37.0% (1421/3838) (Fig. 2c). While taken individually, the highest resistance was observed in the following antibiotics: ampicillin 75.0% (835/1114, 95% CI: 0.72–0.77), amoxicillin 72.7% (322/443, 95% CI: 0.68–0.77), tetracycline 69.1% (498/721, 95% CI: 0.66–0.72), cotrimoxazole 59.3% (1119/1888, 95% CI: 0.57–0.61), nalidixic acid 59.0% (777/1317, 95% 0.56–0.62), cefpodoxime 57.8% (166/287, 95% CI: 0.52–0.63), cephalexin 56.6% (146/258, 95% CI: 0.50–0.63), and cefuroxime 55.2% (389/705, 95% CI: 0.51–0.59). Meanwhile, virtually almost all isolates were susceptible to the carbapenems with the following resistance rates: ertapenem in 0.4% (1/227, 95% CI: 0.00–0.03), imipenem 0.7% (5/567, 95% CI: 0.00–0.02), and meropenem in 0.3% (1/354, 95% CI: 0.00–0.02) (Table 2).

**Table 2:** Meta-analysis of antibiotic resistance for UPEC isolates from urinary tract infections

Antibiotics	No of studies	n/N	Random model		Heterogeneity		Egger’s test		
			% (95% CI)	P	Q	P	I^2^	t	P
Amikacin	8	214/1074	19.9 (0.18–0.22)	< 0.001	344.4	< 0.001	96.5	3.98	0.002
Amoxicillin	3	322/443	72.7 (0.68–0.77)	< 0.001	225.6	< 0.001	94.7	4.76	0.001
Amoxiclav	6	407/998	40.8 (0.38–0.44)	< 0.001	406.2	< 0.001	97.1	2.35	0.039
Ampicillin	8	835/1114	75.0 (0.72–0.77)	< 0.001	222.9	< 0.001	94.6	1.15	0.276
Ampicillin-sulbactam	3	161/354	45.5 (0.40–0.51)	0.089	178.0	< 0.001	93.3	5.54	< 0.001
Aztreonam	2	78/354	22.0 (0.18–0.27)	< 0.001	172.8	< 0.001	93.1	24.1	< 0.001
Cefepime	7	280/952	29.4 (0.27–0.32)	< 0.001	143.3	< 0.001	91.6	3.38	0.006
Cefixime	3	120/443	27.1 (0.23–0.31)	< 0.001	124.0	< 0.001	90.3	5.58	0.001
Cefoperazone-sulbactam	2	36/198	18.2 (0.13–0.24)	< 0.001	81.21	< 0.001	85.2	24.2	< 0.001
Cefotaxime	7	379/1055	35.9 (0.33–0.39)	< 0.001	235.5	< 0.001	94.9	3.99	0.002
Cefoxitin	4	104/707	14.7 (0.12–0.18)	< 0.001	91.61	< 0.001	86.9	13.6	< 0.001
Cefpodoxime	2	166/287	57.8 (0.52–0.63)	0.008	182.7	< 0.001	93.4	11.5	< 0.001
Ceftazidime	9	509/1209	42.1 (0.39–0.45)	< 0.001	212.1	< 0.001	94.3	3.33	0.007
Ceftriaxone	5	247/844	29.3 (0.26–0.32)	< 0.001	239.6	< 0.001	95.0	5.50	< 0.001
Cefuroxime	5	389/705	55.2 (0.51–0.59)	0.006	288.2	< 0.001	95.8	3.16	0.009
Cephalexin	3	146/258	56.6 (0.50–0.63)	0.035	189.3	< 0.001	93.7	12.8	< 0.001
Cephalothin	3	82/437	18.8 (0.15–0.23)	< 0.001	181.0	< 0.001	93.4	3.23	0.008
Cephazolin	3	142/258	55.0 (0.49–0.61)	0.106	168.4	< 0.001	92.9	13.7	< 0.001
Ciprofloxacin	12	792/1781	44.5 (0.42–0.47)	< 0.001	265.5	< 0.001	95.5	0.54	0.602
Ertapenem	1	1/227	0.4 (0.00–0.03)	< 0.001	0.799	1.000	0.00	0.49	0.634
Fosfomycin	1	9/107	8.4 (0.04–0.15)	< 0.001	37.35	< 0.001	67.9	21.0	< 0.001
Gentamicin	13	637/1888	33.7 (0.32–0.36)	< 0.001	269.6	< 0.001	95.6	0.70	0.497
Imipenem	7	5/767	0.7 (0.00–0.02)	< 0.001	3.719	0.988	0.00	5.02	< 0.001
Meropenem	3	1/354	0.3 (0.00–0.02)	< 0.001	1.416	1.000	0.00	2.40	0.035
Nalidixic acid	9	777/1317	59.0 (0.56–0.62)	< 0.001	248.2	< 0.001	95.2	1.70	0.118
Nitrofurantoin	10	297/1486	20.0 (0.18–0.22)	< 0.001	297.1	< 0.001	96.0	3.77	0.003
Norfloxacin	5	286/614	46.6 (0.43–0.51)	0.090	273.1	< 0.001	95.6	3.20	0.009
Ofloxacin	2	101/244	41.4 (0.35–0.48)	0.007	153.5	< 0.001	92.2	13.6	< 0.001
Tetracycline	6	498/721	69.1 (0.66–0.72)	< 0.001	207.3	< 0.001	94.2	2.44	0.033
Tobramycin	1	30/107	28.0 (0.20–0.37)	< 0.001	103.8	< 0.001	88.4	35.2	< 0.001
Co-trimoxazole	13	1119/1888	59.3 (0.57–0.61)	< 0.001	177.1	< 0.001	93.2	1.06	0.313

**Table 3:** Meta-analysis of virulence factors for UPEC isolates from urinary tract infections

Antibiotics	No of studies	n/N	Random model		Heterogeneity		Egger’s test		
			% (95% CI)	P	Q	P	I^2^	t	P
*aer*	3	229/437	52.4 (0.48–0.57)	0.315	189.2	< 0.001	93.1	4.45	0.001
*afa*	5	98/701	14.0 (0.12–0.17)	< 0.001	169.6	< 0.001	92.3	4.54	0.001
*chuA*	1	46/227	20.3 (0.16–0.26)	< 0.001	93.10	< 0.001	86.0	25.9	< 0.001
*cnf1*	5	91/682	13.3 (0.11–0.16)	< 0.001	71.34	< 0.001	81.8	13.2	< 0.001
*Colicin*	1	13/137	9.5 (0.06–0.16)	< 0.001	45.42	< 0.001	71.4	16.9	< 0.001
*CSH*	1	120/150	80.0 (0.73–0.86)	< 0.001	242.1	< 0.001	94.6	39.3	< 0.001
*eco274*	1	99/227	43.6 (0.37–0.50)	0.055	157.9	< 0.001	91.8	33.7	< 0.001
*fimH/MSHA*	10	881/1170	75.3 (0.73–0.78)	< 0.001	210.7	< 0.001	93.8	0.72	0.489
*fimP/MRHA*	4	219/616	35.6 (0.32–0.39)	< 0.001	152.0	< 0.001	91.5	8.02	< 0.001
*fyuA*	1	41/227	18.1 (0.14–0.24)	< 0.001	85.68	< 0.001	84.8	24.8	< 0.001
*hlyA*	12	334/1511	22.1 (0.20–0.24)	< 0.001	241.9	< 0.001	94.6	2.62	0.022
*iucD*	2	130/198	65.7 (0.59–0.72)	< 0.001	203.3	< 0.001	93.6	29.9	< 0.001
*iutA*	2	144/233	61.8 (0.55–0.68)	< 0.001	198.6	< 0.001	93.5	18.9	< 0.001
*kpsMTII*	2	120/198	60.6 (0.54–0.67)	0.003	191.2	< 0.001	93.2	36.4	< 0.001
*PAI*	3	265/480	55.2 (0.51–0.60)	0.023	241.8	< 0.001	94.6	3.80	0.003
*pap*	9	350/1158	30.2 (0.28–0.33)	< 0.001	87.35	< 0.001	98.9	0.54	< 0.001
*sat*	1	28/107	26.2 (0.19–0.35)	< 0.001	100.3	< 0.001	87.0	25.3	< 0.001
*sfa*	5	262/701	37.4 (0.34–0.41)	< 0.001	10.08	< 0.001	90.8	0.05	0.001
*shiA*	1	209/227	92.1 (0.88–0.95)	< 0.001	292.1	< 0.001	95.6	45.4	< 0.001
*sisA*	1	164/227	72.2 (0.66–0.78)	< 0.001	234.0	< 0.001	94.5	40.9	< 0.001
*sisB*	1	56/227	24.7 (0.19–0.31)	< 0.001	106.9	< 0.001	89.8	27.8	< 0.001
*sivH*	1	81/227	35.7 (0.30–0.42)	< 0.001	137.5	< 0.001	90.6	31.5	< 0.001
*traT*	2	266/354	75.1 (0.70–0.79)	< 0.001	236.2	< 0.001	94.5	40.7	< 0.001
*vat*	1	63/227	27.8 (0.22–0.34)	< 0.001	115.9	< 0.001	88.8	28.9	< 0.001
*yfcv*	1	57/227	25.1 (0.20–0.31)	< 0.001	108.2	< 0.001	88.0	27.9	< 0.001

Regarding the virulence factors, both factors associated with *E. coli* surface cell and those secreted and exported to the site of action were observed. Taking into account the groups of virulence factors according to their action mechanisms, a high prevalence was observed among immune suppressors in 54.1% (874/1615), followed by adhesins in 45.9% (2316/5048), siderophore systems in 41.8% (647/1549) and toxins in 19.9% (529/2664) (Fig. 2d). Taken individually, the most prevalent virulence factors from adhesins group were: the cell surface hydrophobicity (CSH) in 80% (120/150, 95% CI: 0.73–0.86), the fimbrial and afimbrial adhesins: *fimH/MSHA* in 75.3% (881/1170, 95% CI: 0.73–0.78), *fimP/MRHA* in 35.6% (219/616, 95% CI: 0.32–0.39), the serum resistance coded by the gene *traT* in 75.1% (266/354, 95% CI: 0.70–0.79), the capsular polysaccharide K antigen (kpsMTII) in 60.6% (120/198, 95% CI: 0.54–0.67) and *pap* in 30.2% (350/1158, 95% CI: 0.28–0.33). Frequencies of immune suppressors coded by the pathogenicity islands (PAIs) genes were *shiA* in 92.1% (209/227, 95% CI: 0.88–0.95), *sisA* in 72.2% (164/227, 95% CI: 0.66–0.78), *sisB* in 24.7% (56/227, 95% CI: 0.19–0.31) and *PAI* in 55.2% (265/480, 95% CI: 0.51–0.60). The secreted virulence factors exported to the site of infection were represented by toxins and siderophore molecules. The most frequent toxins observed were the haemolysin (*hlyA*) in 22.1% (334/1511, 95% CI: 0.20–0.24), the secreted autotransporter toxin (*sat*) in 26.2% (28/107, 95% CI: 0.19–0.35) and the cytotoxic necrotizing factor-1 (*cnf*-1) in 13.3% (91/682, 95% CI: 0.11–0.16). For siderophores, the aerobactin system was observed most frequently, and included outer membrane proteins genes: *iucD* in 65.7% (95% CI: 0.59–0.72), *iutA* in 61.8% (0.55–0.68), the aerobactin (*aer*) in 52.4% (130/198, 95% CI: 0.48–0.57) and the heme receptor genes (*chuA*) in 20.3% (46/227, 95% CI: 0.16–0.26) (Table 3). High rates of AMR and virulence factors are statistically significant among in-patients than in out-patients as shown in Table 4.

**Table 4:** Distribution of antibiotics resistance and virulence factors among in- and out-patients

	In-patient, n/N (%)	Out-patient, n/N (%)	OR (95% CI)	*p*-value
**A. Main antibiotic groups**				
Aminoglycosides	403/1432 (28.1)	478/1637 (29.2)	0.96 (0.83–1.12)	0.631
Sulfonamides	610/1021 (59.7)	509/867 (58.7)	1.02 (0.88–1.18)	0.817
Cyclines	416/614 (67.8)	82/107 (76.6)	0.88 (0.65–1.21)	0.441
Fosfomycin	0	9/107 (8.4)	11.3 (0.21–603.0)	0.232
Nitrofurans	130/952 (13.7)	167/534 (31.3)	0.44 (0.34–0.56)	< 0.0001
Quinolones	1333/2217 (60.1)	623/1739 (35.8)	1.68 (1.50–1.88)	< 0.0001
Beta-lactams	2244/5622 (39.9)	2166/6342 (34.2)	0.86 (0.80–0.92)	< 0.0001
**B. Beta-lactams classes**				
Aminopenicillins	846/1117 (75.7)	311/440 (70.7)	1.07 (0.90–1.27)	0.427
Carbapenems	2/659 (0.3)	5/689 (0.7)	0.41 (0.8–2.16)	0.298
Monobactams	77/150 (51.3)	1/204 (0.5)	104.7 (14.4–761.4)	< 0.0001
Beta-lactam+Inhibitors	273/754 (36.2)	331/796 (41.6)	0.87 (0.72–1.05)	0.150
Cephalosporins	1046/2942 (35.6)	1518/4213 (36.0)	1.01 (0.93–1.11)	0.776
**C. Cephalosporins classes**				
1GCs	210/491 (42.8)	160/462 (34.6)	1.24 (0.97–1.57)	0.087
2GCs	118/518 (22.8)	375/894 (41.9)	0.54 (0.43–0.69)	< 0.0001
3GCs	571/1648 (34.7)	850/2190 (38.8)	0.89 (0.89–1.01)	0.072
4GCs	147/285 (51.6)	133/667 (19.9)	2.59 (1.97–3.40)	< 0.0001
**D. Virulence factors groups**				
Adhesins	1635/3309 (49.4)	681/1739 (39.2)	1.3 (1.13–1.40)	< 0.0001
Siderophore systems	276/428 (64.5)	371/1121 (33.1)	1.9 (1.61–2.36)	< 0.0001
Toxins	312/1418 (22.0)	217/1246 (17.4)	1.3 (1.05–1.53)	0.016
Immune suppressors	200/276 (72.5)	674/1339 (50.3)	1.4 (1.17–1.77)	0.001

### Relationship between antimicrobial resistance and virulence factors in UPEC

In this section, we will briefly review the possible relation between AMR and virulence factors in UPEC on selected examples, focusing on resistance to quinolones and beta-lactams. We will discuss how harbouring virulence factors may increase or decrease the possibility of UPEC to develop resistance to antibiotics, although only aggregate data were available and trends in AMR and virulence factor carriage could not be directly related in this analysis.

Previous studies on UPEC reported that quinolone-resistant isolates encoded for virulence factor genes related to their ability to invade the urinary tract [31]. The relevant virulence factors, like haemolysin, aerobactin, cytotoxic necrotizing factor-1 (*cnf*-1) and *sat* are chromosomally encoded in the PAIs, which can be deleted from the chromosome spontaneously and easily [32, 33]. Quinolones can act by increasing the deletion and transposition of DNA regions during the development of quinolone-resistance facilitated by an exposure to quinolones [34]. While PAIs share some characteristics with bacteriophages, it has been proven that pro-phages hidden within chromosomal DNA are excised by the activation of SOS [35], a DNA repair mechanism. Quinolones likely contribute to the partial or total excision of PAIs in a SOS-dependent way because the antimicrobial agents activate the SOS system [36]. Hence, this may induce the loss of virulence factors of quinolone-resistant *E. coli* that are less able to cause invasive UTIs as this phenomenon may result in phenotypic changes in bacteria. Nevertheless, the fact that quinolone-resistance impairs the ability of UPEC to invade local tissue of the kidney and prostate does not disrupt a strain’s capacity to cause bacteraemia (urosepsis) once local invasion has taken place [31].

In *E. coli*, the majority of virulence associated plasmids belong to the F incompatibility group and are often key determinants of antimicrobial resistance [37]. It is conceivable that genetic determinants of virulence may be co-mobilized under antimicrobial selective pressure if they are located on the same genetic platform as antimicrobial resistance genes (plasmids, transposons, integrons) [38]. The relationship between resistance and virulence remains uncertain and depends on the interaction between the strain’s phylogenetic group and the type of resistance determinant [39]. In *Enterobactericeae*, the IncF plasmid family is very widespread and can encode aerobactin as well as other factors of putative virulence such as the *traT* virulence protein, responsible for serum resistance in *E. coli*. Extended-spectrum beta-lactamase (ESBL) producing *E. coli* are emerging and are posing challenges to the clinicians on therapeutic choices; and F-plasmids often encode for ESBL genes from the CTX-M, TEM or SHV groups, as well as genes conferring resistance to other antibiotic groups [40–43]. These few examples demonstrate how antimicrobial pressure can select for plasmids carrying virulence and resistance determinants, and hence allow virulent traits to be selected for by antimicrobial use in a bacterial population.

Some specific lineages within the *E. coli* species, such as the phylogroup B2, show high frequency of virulence factors [44–46]. Independent predictors for pathogenicity have been identified to be alpha-hemolysin, yersiniabactin receptor (*fyuA*), serum resistance-associated outer membrane protein (*traT*), and aerobactin receptor type *iutA*. In strains producing the *bla*_CTX-M-1_ and *bla*_CTX-M-9_ group ESBL enzymes, respectively, *iutA* and *traT* were significantly more common among these virulence factors [47]. Similar results, where *iut* and *traT* are more prevalent, have been reported in *E. coli* CTX-M ESBL group from UTIs [48].

## Discussion

Uropathogenic *Escherichia coli* (UPEC) are the primary bacterial type associated with urinary tract infection (UTI) [1]. They include diverse *E. coli* phylogroups that express a wide range of virulence factors and genes that can increase its pathogenicity and resistance to antimicrobials [4, 49–52]. During the last few decades, the emergence of high rates of antimicrobial resistance and multidrug resistance (MDR) phenotype reported in UPEC has become a major concern worldwide [53, 54]. In this study, we reported virulence factors and antimicrobial resistance of UPEC.

The study of AMR showed variable proportions of resistance in different antimicrobial categories. High resistance rates were observed in aminopencillins, tetracyclins, cotrimoxazole, nalidixic acid and cephalopsporins. Several studies have reported high resistance rates of UPEC on these antibiotics and by different mechanisms [52, 53, 55, 56]. This study showed high resistance to beta-lactam antibiotics. The increasing rate of 3rd-generation cephalosporin resistance, suggesting extended-spectrum beta-lactamase (ESBL) producing *E. coli* is of concern worldwide. It has been reported that carbapenems are the best options for treating ESBL UPEC-producers [1, 57], and our findings report similar results with susceptibility rates to carbapenems close to 100%. However, there is high risk of a similar development like the spread of ESBL following the widespread use of 3rd generation cephalosporins; the spread of carbapenem resistance mechanisms if these are used routinely. Using carbapenems as first-line antimicrobial treatment does not make them the best option as first line over oral agents like nitrofurantoin and/or fosfomycin in treating UTIs, and reserving carbapenem use for extensively drug resistant isolates with few or no other treatment alternatives.

Regarding virulence factors of UPEC, this study showed a high prevalence of fimbriae (*fimH/MSHA:* 75%). P fimbriae and type 1 fimbriae are known to play a key role in the pathogenesis by facilitating the attachment of *E. coli* to the uroepithelium [58]. The *fimH* adhesion mediates the adherence of UPEC to the bladder epithelium as well as the invasion of bladder epithelial and mast cells into caveolae, which has been reported to protect the bacteria from host defences and antimicrobials [59]. In addition to that, the P-fimbrial adhesins, encoded by the *papG* gene, mediate the attachment to the P-blood group antigens on uroepithelial cells [59]. The expression of *E. coli* surface adhesins is increased by initiating the close contact of the bacteria with the host cell wall. Receptors for S- and P-fimbriae are located, in UPEC pathotypes, on the surface of epithelial cells lining the host urinary tract [10], and the high hydrophobicity of bacterial cell promotes the adherence of UPEC to mucosal epithelial cells surfaces [60]. UPEC pathotypes carry significantly higher numbers of fimbrial gene clusters compared to faecal/commensal pathotypes [61]. Siderophores bind ferric iron and iron-siderophore complexes are recognised by cognate outer-membrane receptors. UPEC pathotypes encode the proteins required for the biosynthesis and uptake of several siderophores, such as enterobactin, aerobactin, yersiniabactin and salmochelin [61]. Haemolysin and siderophores are secreted virulence factors that enable the UPEC to colonize the urinary tract and persist despite the effectively functioning host immune defence mechanism [53]. The iron uptake systems of UPEC are mediated by the siderophore aerobactin synthesized by a number of *iuc* genes and proteins encoded by *iut* genes mediate its transport [62, 63]. This study showed prevalence of *iucD* and *iutA* genes of 66 and 62%, respectively. The toxins produced by UPEC inflict tissue damage and are involved in the host-pathogen interplay [61]. This is mediated by the haemolysin (*hlyA*), in addition to its cytolytic effect. The *hlyA* was the most reported toxin in this review, followed by *sat* and *cnf*-*1*. The *cnf-1* help the UPEC to survive even in the presence of neutrophils [61]. However, the invasins like the *sisA* and *sisB* play a key role in suppressing the host immune response during the initial stages of infection [64]. The summary of UPEC virulence factors mechanisms is in Table 5. Virulence factors and antimicrobial resistance patterns of UPEC is varying from a region to another. A local and/or national antimicrobial resistance and UPEC virulence factors study may be useful for staying abreast regarding the trend for the UTIs’ empirical treatment [9]. Intervention strategies on virulence factors that govern the UPEC-mediated UTIs symptomatology may protect against a wide range of UTI syndromes.

**Table 5:** UPEC virulence factors mechanisms of action

Virulence factors groups	Examples of genes	Mechanisms
Adhesins	*afa, CSH, fimH, fimP, kpsmtII, pap, sfa, traT*	UPEC adhesins can contribute to virulence in different ways: (i) directly triggering host and bacterial cell signalling pathways, (ii) facilitating the delivery of other bacterial products to host tissues, and (iii) promoting bacterial invasion [3]. Adhesins help in the adhesion of organism to epithelial cell surface, thereby it escapes from flushing action during micturition [7]. Fimbriae is responsible for adhesion, colonization, invasion of host epithelium and makes UPEC to escape from the innate immune system by internalization process within urothelial cells which is mediated by the transduction cascades [8].
Toxins	*Cnf1, hlyA, saT, vaT*	Toxins like haemolysin and Cytotoxic Necrotising Factor (CNF) act by their cytotoxicity and invasiveness. Haemolysin production could inhibit the cytokine production of host cells and promote the cytotoxicity. It causes lysis of the erythrocytes which release nutrients and other vitamins available for the bacteria. At the same time it releases inflammatory mediators and enzymes which are cytotoxic to renal proximal tubular epithelial cells, erythrocytes and leukocytes, thereby causing renal epithelial damage [3]. CNF interferes with the phagocytosis of *E. coli* by the WBCs and thus it leads to exfoliation and apoptosis of bladder epithelial cells. It further enhances the easy access of bacteria into the underlying tissue. These toxins can alter signalling pathways, provoke the inflammatory response and prevent the apoptosis thereby they cause the UPEC population to expand [1].
Siderophores	*aer, chuA, fyuA, iuD, iutA, yfcv*	Production of siderophores by *E. coli* which takes up iron from the host and helps in colonization and survival of pathogen [1, 8]. They contribute to the process of nutritional passivation of metal ions, in which UPEC access these vital nutrients while simultaneously protecting themselves from their toxic potential [65]
Immune suppressors	*PAI, shiA, sisA, sisB, sivH, Eco274*	UPEC induces a non-sterilizing adaptive immune response in the bladder. Its causes long-lasting changes in the bladder urothelium, conferring resistance or increased susceptibility to subsequent infections depending on the outcomes of the first infection [66]. The invasins play a key role in suppressing the host immune response during the initial stages of infection [64].

## Conclusion

Relative high rates in antimicrobial resistance were observed among aminopenicillins, beta-lactams associated with inhibitors, tetracyclines, sulfonamides, quinolones and 1st generation cephalosporins. This suggests a reassessment of empirical therapies in urinary tract infections treatment caused by this pathogen. The most frequent observed virulence factors included both surface and secreted virulence factors (*shiA*, *CSH*, *fimH/MSHA*, *traT*, *sisA*, *iucD*, *iutA*, *kpsMTII*, and *PAI*).

## Supplementary Information

**Additional file 1.** Newcastle-Ottawa Scale adapted for cross-sectional studies.

**Additional file 2: Table S1.** Studies assessment using the Newcastle-Ottawa Scale adapted for assessment of cross-sectional studies.

## Data Availability

All data generated or analysed during this study are included in this published article.

## Abbreviations

UPEC: Uropathogenic *Escherichia coli*
AMR: Antimicrobial resistance
UTI: Urinary tract infection
MGE: Mobile genetic elements
ExPEC: Extra-intestinal pathogenic *E. coli*
PAIs: Pathogenicity islands
MDR: Multidrug resistance
PRISMA: Preferred reporting items for systematic reviews and meta-analyses
NOS: Newcastle-Ottawa Scale
ESBL: Extended-spectrum beta-lactamase
