# Supplementary material for: Virulence factors and antimicrobial resistance of uropathogenic Escherichia coli (UPEC) isolated from urinary tract infections: a systematic review and meta-analysis
Source: BMC Infect Dis. 2021 Aug 4;21:753. doi: 10.1186/s12879-021-06435-7 (PMC8336361; doi:10.1186/s12879-021-06435-7)
Supplement: Supplementary file 1 — Additional file 1. Newcastle-Ottawa Scale adapted for cross-sectional studies. [file 12879_2021_6435_MOESM1_ESM.docx]

**Additional file 1:**

**Newcastle-Ottawa Scale adapted for cross-sectional studies**

**Selection:**

1. Representativeness of the sample:
   1. Truly representative of the average in the target population. * (all subjects or random sampling)
   2. Somewhat representative of the average in the target group. * (non-random sampling)
   3. Selected group of users/convenience sample.
   4. No description of the derivation of the included subjects.
2. Sample size:
   1. Justified and satisfactory (including sample size calculation). *
   2. Not justified.
   3. No information provided
3. Non-respondents:
   1. Proportion of target sample recruited attains pre-specified target or basic summary of non-respondent characteristics in sampling frame recorded. *
   2. Unsatisfactory recruitment rate, no summary data on non-respondents.
   3. No information provided
4. Ascertainment of the exposure (risk factor):
   1. Vaccine records/vaccine registry/clinic registers/hospital records only. **
   2. Parental or personal recall and vaccine/hospital records. *
   3. Parental/personal recall only.

**Comparability:** (Maximum 2 stars)

1. Comparability of subjects in different outcome groups on the basis of design or analysis. Confounding factors controlled.
   1. Data/ results adjusted for relevant predictors/risk factors/confounders e.g. age, sex, time since vaccination, etc. **
   2. Data/results not adjusted for all relevant confounders/risk factors/information not provided.

**Outcome:**

1. Assessment of outcome:
   1. Independent blind assessment using objective validated laboratory methods. **
   2. Unblinded assessment using objective validated laboratory methods. **
   3. Used non-standard or non-validated laboratory methods with gold standard. *
   4. No description/non-standard laboratory methods used.
2. Statistical test:
   1. Statistical test used to analyse the data clearly described, appropriate and measures of association presented including confidence intervals and probability level (p value). *
   2. Statistical test not appropriate, not described or incomplete.

Cross-sectional Studies:

Very Good Studies: 9-10 points

Good Studies: 7-8 points

Satisfactory Studies: 5-6 points

Unsatisfactory Studies: 0 to 4 points

This scale has been adapted from the Newcastle-Ottawa Quality Assessment Scale for cohort studies to provide quality assessment of cross sectional studies^^[[1]](#footnote-1)^^.

1. Herzog R, et al. Is Healthcare Workers’ Intention to Vaccinate Related to their Knowledge, Beliefs and Attitudes? A Systematic Review. *BMC Public Health* 2013 **13**:154 [↑](#footnote-ref-1)
