# Supplementary material for: Virulence factors and antimicrobial resistance of uropathogenic Escherichia coli (UPEC) isolated from urinary tract infections: a systematic review and meta-analysis
Source: BMC Infect Dis. 2021 Aug 4;21:753. doi: 10.1186/s12879-021-06435-7 (PMC8336361; doi:10.1186/s12879-021-06435-7)
Supplement: Supplementary file 2 — Additional file 2: Table S1. Studies assessment using the Newcastle-Ottawa Scale adapted for assessment of cross-sectional studies. [file 12879_2021_6435_MOESM2_ESM.docx]

**Additional file 1:**

**Table S1: Studies assessment using the Newcastle-Ottawa Scale adapted for assessment of cross-sectional studies**

| Studies | Representativeness | Sample size | Non-respondents | Ascertainment of the exposure | Comparability | Assessment of outcome | Statistical test | Total | Categories |
| --- | --- | --- | --- | --- | --- | --- | --- | --- | --- |
| Ghazvini et al., 2019 (1) | 1 | 0 | 0 | 2 | 2 | 2 | 1 | 8 | Good Studies |
| Ghazvini et al., 2019 (2) | 1 | 0 | 0 | 2 | 0 | 2 | 1 | 6 | Satisfactory Studies |
| Jadhav et al., 2011 | 1 | 0 | 0 | 2 | 0 | 2 | 1 | 6 | Satisfactory Studies |
| Kot et al., 2016 | 1 | 0 | 0 | 2 | 0 | 2 | 1 | 6 | Satisfactory Studies |
| Malekzadegan et al., 2018 | 1 | 0 | 0 | 2 | 2 | 2 | 1 | 8 | Good Studies |
| Miranda-Estrada et al., 2017 | 1 | 0 | 0 | 2 | 2 | 2 | 1 | 8 | Good Studies |
| Neamati et al., 2015 | 1 | 0 | 0 | 2 | 0 | 2 | 0 | 5 | Satisfactory Studies |
| Oliveira et al., 2011 | 1 | 0 | 0 | 2 | 2 | 2 | 1 | 8 | Good Studies |
| Olorunmola et al., 2013 | 1 | 0 | 0 | 2 | 0 | 2 | 0 | 5 | Satisfactory Studies |
| Raeispour et al., 2018 | 1 | 0 | 0 | 2 | 0 | 2 | 0 | 5 | Satisfactory Studies |
| Shakhatreh et al., 2019 | 1 | 0 | 0 | 2 | 0 | 2 | 0 | 5 | Satisfactory Studies |
| Tabasi et al., 2015 | 1 | 0 | 0 | 2 | 2 | 2 | 1 | 8 | Good Studies |
| Wang et al., 2014 (1) | 1 | 0 | 0 | 2 | 2 | 2 | 1 | 8 | Good Studies |
| Wang et al., 2014 (2) | 1 | 0 | 0 | 0 | 2 | 2 | 1 | 6 | Satisfactory Studies |
